# Cardiac dysfunctions following spinal cord injury

**Published:** 2009-04-25

**Authors:** VT Grigorean, AM Sandu, M Popescu, MA Iacobini, R Stoian, C Neascu, F Popa

**Affiliations:** * ‘Bagdasar–Arseni’Clinical Emergency Hospital, Department of General Surgery, Bucharest, Romania; ** ‘Bagdasar–Arseni’Clinical Emergency Hospital, Department of Neurosurgery, Bucharest Romania; *** District Hospital Pitesti, Department of Neurosurgery, Pitesti, Romania; **** ‘Carol Davila’University of Medicine and Pharmacy Bucharest, Romania

**Keywords:** spinal shock, neurogenic shock, cardiac dysrrhythmias, bradycardia, tachyarrhytmias, autonomic dysreflexia, cardiac deconditionting, coronary heart disease

## Abstract

The aim of this article is to analyze cardiac dysfunctions occurring after spinal cord injury (SCI). Cardiac dysfunctions are common complications following SCI. Cardiovascular disturbances are the leading causes of morbidity and mortality in both acute and chronic stages of SCI.

We reviewed epidemiology of cardiac disturbances after SCI, and neuroanatomy and pathophysiology of autonomic nervous system, sympathetic and parasympathetic.

SCI causes disruption of descendent pathways from central control centers to spinal sympathetic neurons, originating into intermediolateral nuclei of T1–L2 spinal cord segments.  Loss of supraspinal control over sympathetic nervous system results in reduced overall sympathetic activity below the level of injury and unopposed parasympathetic outflow through intact vagal nerve.

SCI associates significant cardiac dysfunction. Impairment of autonomic nervous control system, mostly in patients with cervical or high thoracic SCI, causes cardiac dysrrhythmias, especially bradycardia and, rarely, cardiac arrest, or tachyarrhytmias and hypotension. Specific complication dependent on the period of time after trauma like spinal shock and autonomic dysreflexia are also reviewed. Spinal shock occurs during the acute phase following SCI and is a transitory suspension of function and reflexes below the level of the injury. Neurogenic shock, part of spinal shock, consists of severe bradycardia and hypotension. Autonomic dysreflexia appears during the chronic phase, after spinal shock resolution, and it is a life–threatening syndrome of massive imbalanced reflex sympathetic discharge occurring in patients with SCI above the splanchnic sympathetic outflow (T5–T6). Besides all this, additional cardiac complications, such as cardiac deconditioning and coronary heart disease may also occur.

Proper prophylaxis, including nonpharmacologic and pharmacological strategies and cardiac rehabilitation diminish occurrence of the cardiac dysfunction following SCI. Each type of cardiac disturbance requires specific treatment.

Spine cord injury (SCI) is a real health problem, being one of the most devastating of all traumatic events.

The aim of this article is evaluation of cardiac disturbances following SCI. Cardiac dysfunctions are common consequences of SCI and they represent an issue of interest worldwide. In SCI important cardiovascular dysfunctions occur during acute and chronic stages. [1] Cardiovascular disturbances are the leading causes of morbidity and mortality in both acute and chronic stages of SCI.[2,4] Heart disease is the most common cause of death, accounting for approximately 30% of deaths.[3]

Impairment of autonomic nervous control system, mostly in patients with cervical or high thoracic SCI, causes cardiac dysrrhythmias, especially bradycardia and rarely cardiac arrest or tachyarrhytmias, and hypotension. Specific complication dependents on the period of time after trauma like spinal shock occurring during the acute phase and autonomic dysreflexia found mostly during the chronic phase. Besides, additional cardiac complications, such as cardiac deconditioning and coronary heart disease may also occur. 

## Epidemiology

SCI has an annual incidence of 15 to 52.5 cases per million population, and about 80% are young males, aged between 15 and 35 years, and 5% are children.[1,5–9] Neurological disability is frequently encountered, from all patients 53% are tetraplegic and 42% are paraplegic.

Proper treatment of cardiovascular dysfunctions pays an important role in the therapeutically management of SCI. By diminishing the cardiovascular complications, morbidity and mortality are reduced and the quality of life is raised. Cardiovascular dysfunctions are the second most common underlying cause of death in chronic SCI, with a frequency of 21.6%, after neoplasms. They contribute to death in 18.9% of cases. Taking both, underlying and contributing causes, cardiovascular dysfunctions are responsible for 40.5% of deaths, being the most common cause of mortality in patients with SCI. [3] The most common causes of death are cardiac failure, ventricular tachycardia, atria fibrillation, cardiac arrest, atherosclerosis and coronary heart disease, cerebrovascular disease, rupture of abdominal aneurysm, cardiomyopathy and ill–defined heart disease. [3] 

100% of patients sustaining complete motor cervical SCI (ASIA A and B) have bradycardia and 16% develop cardiac arrest. [1] In incomplete motor cervical SCI (ASIA C and D), 35–71% develop bradycardia. [1] Extremely rare they have cardiac arrest. [1] In thoracolumbar SCI in only 13–35% of cases bradycardia occurs. [1] 

Besides, in this particular group of patients, lack of physical activity, obesity, hyperlipidemia, insulin resistance, and diabetes have a higher frequency in patients with SCI than in the general population. [1]

## Neuroanatomy

Medial prefrontal cortex, insula, hypothalamus, and cuneiform nucleus project into cardiovascular nuclei within medulla oblongata. [10–12] Parasympathetic impulses are carried out via vagus nerve. Preganglionic fibers synapse with postganglionic parasympathetic neurons near myocardium. Peripheral vessels, there is no parasympathetic outflow.

Regarding the sympathetic activity, preganglionic neurons are situated within the lateral horn into intermediolateral nuclei of T1–L2 cord segments. Heart sympathetic impulses arise from T1–T4 spinal cord segments. Sympathetic preganglionic neurons exit the spinal cord via ventral root. They synapse with postganglionic neurons situated into the paravertebral sympathetic chain. Postganglionic sympathetic fibers outflow through peripheral nerves to heart and vessels.

## Pathophysiology

Besides well–known motor and sensitive deficits, in SCI, autonomic disturbances are frequently encountered. Following SCI often occur a dysfunction in communication between brainstem and autonomic nervous system. Autonomic nervous system pays a highly important role in cardiovascular control. Impulses from autonomic nervous system, sympathetic and parasympathetic, control heart rate, and blood pressure. Sympathetic and parasympathetic nervous systems have antagonistic effects, adapted to various needs. Parasympathetic decreases heart rate. Sympathetic increases heart rate, myocardic contractibility, raises peripheral vascular resistance and blood pressure, by inducing vasoconstriction. Heart rate and blood pressure control depends on the activity of supraspinal centers, which send activator impulses, through descendent pathways, to sympathetic spinal preganglionar neurons. 

Secondary to SCI descendent pathways are interrupted and spinal circuits become unable to generate sympathetic activity. Disruption of descendent pathways results in sympathetic hypoactivity and unopposed parasympathetic outflow through intact vagal parasympathetic control. Sympathetic hypoactivity results in low heart rate, reflex bradycardia and rarely cardiac arrest, low resting blood pressure, orthostatic hypotension, loss of regular adaptability, loss of diurnal fluctuation of blood pressure and disturbed reflex control. Because heart sympathetic impulses arise from T1–T4 spinal cord segments, complete cervical and high thoracic SCI interferes with superior centers control over spinal sympathetic activity to the heart. 

Besides autonomic imbalance immediately after the trauma, a direct injury of the myocardium occurs due to massive adrenergic mediators releasing from the suprarenal glands and sympathetic nerves. 

As subsequent consequences to loss of sympathetic activity, some morphological changes arise into preganglionic sympathetic neurons below the injury level and a hyperresponsiveness of peripheral alpha–adrenergic receptors are encountered. It is not established yet whether alpha–adrenergic receptor hyperresponsiveness is a consequence of receptor hypersensitivity or insufficient presynaptic uptake of the norepinephrine.[13]

## Cardiac dysfunctions following SCI

During the acute phase of SCI patients develop spinal shock with neurogenic shock.  Autonomic dysreflexia and coronary heart disease are encountered during the chronic phase of SCI. Cardiac dysrrhythmias: bradyarrhythmias, such as bradycardia or asystola, or tachyarrhythmias, such as paroxistic supraventricular tachycardia, sinus tachycardia, atria flutter and atria fibrillation, and cardiac deconditioning occur in both acute and chronic stages. 

### Cardiac dysfunctions occurring during the acute phase of SCI

The initial response to SCI is a short period of massive sympathetic stimulation and reflex parasympathetic activity that usually lasts for 3 to 4 minutes, mediated by alpha–adrenergic receptors. The patient develops reflex bradycardia or tachyarrhythmias and severe hypertension. The sympathetic stimulation is due to massive norepinephrine releasing from the suprarenal glands and disruption of cervical and high thoracic vasoactive neurons.[14] 

After this immediate response to trauma, occurs decrease in sympathetic activity, due to interruption of descendent sympathetic pathways. Cardiac output and total peripheral resistance decrease, while central venous pressure remains unchanged. The patient is prone to bradycardia, hypotension, and hypothermia, by lack of sympathetic activity and unopposed vagal tone.

#### Spinal shock

The interruption of the communication between the superior centers and peripheral sympathetic intermediolateral thoracic and lumbar neurons leads to spinal shock.

Spinal shock accompanies SCI during the acute phase and is a physiological disruption of all functions of the spinal cord. It is a transitory suspension of function and reflexes below the level of the injury. Activity of sympathetic spinal neurons is modulated by inputs from autonomic central centers. After trauma occurs a sudden disruption of communication between these centers and the sympathetic neurons in the intermediolateral thoracic and lumbar spinal cord leading to spinal shock.

Spinal shock was first described by Whytt in 1750, and the term of ‘spinal shock’ was later introduced into the literature by Hall in 1841.[15] The features of spinal shock are sensory deficits, flaccid paralysis, absence of deep tendon reflexes, abolishment of reflex somatic activity, and thermoregulatory disturbances below the level of injury. Etiology and pathogenesis of the spinal shock remain controversial. There are different theories that partially explain the phenomenon. [15, 16] Spinal shock involves different aspects according to the level of cord injury. In high cervical SCI occurs acute respiratory failure, tetraplegia, anesthesia, lack of all reflexes below injury site, neurogenic shock, detrusor and rectum areflexia, ipsilateral Horner syndrome (ptosis, enophthalmos, miosis, anhidrosis). In low cervical SCI there is no respiratory failure, because respiratory muscles are not affected. Patients with high thoracic SCI develop paraparesis. In low thoracic injuries there is no arterial hypotension and no neurogenic shock.[17] 

Neurogenic shock consists of bradycardia, severe arterial hypotension and hypotermia.[17–20]  It is a consequence of autonomic nervous system malfunction, and it is caused by the lack of sympathetic activity, through loss of supraspinal control and unopposed parasympathetic tone via intact vagus nerve. [18–21] Systolic blood pressure below 90 mmHg in resting position, which is not a result to low intravascular volume due to hemorrhage or dehydration, is characteristic for neurogenic shock. Severe arterial hypotension usually requires vasopressors.[18, 19,22] Severity of arterial hypotension and need for vasopressors are directly proportionate with the severity of the injury.[23] Cardiac output and peripheral vascular resistance deceases, while central venous pressure remains unchanged.[14] Spinal shock usually lasts days to weeks, with an average of 4–12 weeks. [15,17–19,22,24–27] There is no consensus regarding clinical signs that defines the duration of spinal shock. Appearance of bulbocavernous reflex, recovery of deep tendon reflexes or return of reflex detrusor functions are considered by different authors the endpoint of spinal shock. Ditunno et al. believe that spinal shock consists of four phases: areflexia or hyporeflexia (0–24 hours), initial reflex return (1–3 days), early hyperreflexia (4 days–1 month) and spasticity (1–12 months).[28] 

Neurogenic shock should not be mistaken with hypovolemic shock. In neurogenic shock, hypotension is associated with bradycardia, while in hypovolemic shock tachycardia occurs. In neurogenic shock the skin is warm and dry, except for patients exposed to a cold environment. Care should be taken because neurogenic and hypovolemic shock may coexist, and when this happens, neurogenic shock exacerbates the effects of hypovolemic shock by disabling the vasoconstrictive reflexes that ordinarily preserve blood flow to vital organs.

### Cardiac dysfunctions occurring in both acute and chronic phase of SCI

#### Cardiac dysrrhythmias

The risk of cardiac dysrrhythmias is higher during the acute phase and diminishes with time passing. Cardiac dysfunctions are usually life–threatening during the first few weeks after the injury.[18] Even though, late cardiac dysfunctions may occur during the chronic phase. 

Cardiac dysrrhythmias includes:

- Bradyarrhythmias
  - Bradycardia
  - Asystola, cardiac arrest
- Tachyarrhythmias
  - Paroxistic supraventricular tachycardia
  - Sinus tachycardia
  - Atria flutter
  - Atria fibrillation

Ventricular bradyarrythmias are the most frequent cardiac dysfunctions found following SCI in both acute and chronic phase. Parasympathetic outflow, via vagus nerve, remains intact, inducing reflex bradycardia, especially in acute cervical SCI, and rarely cardiac arrest.[1,18] 

All this, plus hypotension lead to hemodynamic instability.[29– 31] Usually bradycardia and cardiac arrest occurring during the acute phase following SCI are temporary.[32,33] The frequency of bradyarrhythmias peaked on day 4 after injury and gradually declined thereafter. After the acute phase, the risk for arrhythmia diminishes.

During the chronic phase of SCI, incidence of bradyarrhythmia is higher in tetraplegics and is rarely found in paraplegics. [18,32–35] Late asystole requiring transvenous ventricular pacing was found during the chronic phase.[34]

Dysrrhythmias, particularly atria fibrillation, may also occur during episodes of autonomic dysreflexia in high SCI. It require immediate pharmacological intervention to restore the normal rhythm.[18, 36,37]

Bradycardia is found in 64–77% cases in cervical SCI. It is more frequently encountered in the acute phase, and is more severe in the first 2–6 weeks after trauma.[1,18] Furlan et al. found that bradycardia and hypotension, occurring immediately after trauma, persist in patients with severe autonomic cardiovascular pathways impairment. Patients with less severe pathways impairment have a higher ventricular rate, although an abnormal heart rate is observed.[23] 

Bradycardia is any heart rate less than 60 beats/minute.

Grading of severity in bradycardia is the following: Mild bradycardia: the patient is asymptomatic, systolic arterial blood pressure is maintained over 90 mmHg, without medication Moderate bradycardia: requires medical intervention for increasing heart rate or for maintain adequate blood pressure Severe bradycardia: asystola

**Fig 1:**
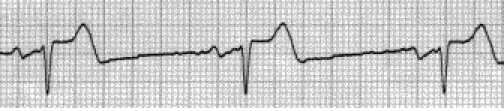
Bradycardia due to an unopposed vagal activity in SCI above T1.

**Fig 2:**
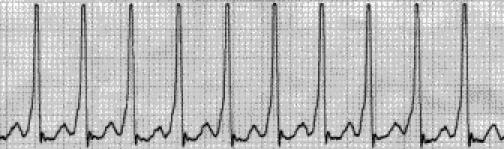
Paroxistic supraventricular tachycardia.

**Fig 3:**
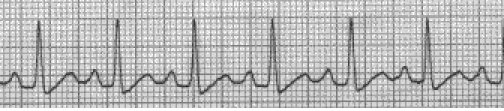
Sinus tachycardia.

**Fig 4:**
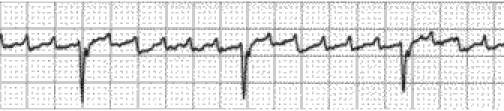
Atria flutter.

**Fig 5:**
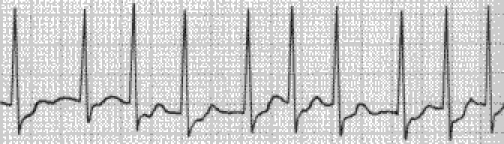
Atria fibrilation.

On EKG bradycardia is most commonly found (Fig 1). Tacharrymias such as paroxistic supraventricular tachycardia (Fig 2), sinus tachycardia (Fig 3), atria flutter (Fig 4) and atria fibrillation (Fig 5) may also be encountered.

Patients with SCI are prone for developing nonspecific changes on EKG, like ST segment elevation. Besides, they may also develop other electric abnormalities, such as premature atria contractions, intraventricular conduction delay, bundle–branch block. [38] 

Tracheal suction, endotracheal intubation, laryngoscopy and other tracheal stimuli may induce cardiac bradycardia and even asystola, especially in the presence of hypoxia.[39]  Reflex bradycardia and cardiac arrest occur due to a vago–vagal reflex. In normal conditions, this reflex is opposed by sympathetic activity. During hypoxia a pulmonary–vagal reflex occurs. It increases breathing rate and pulmonary inflation, as a response to hypoxia. In patients with SCI compensatory sympathetic activity is hindered. Pulmonary–vagal reflex activity is also hindered, because pulmonary inflation cannot increase with hypoxia during mechanical ventilation.[32,33, 40] Bradycardia or cardiac arrest as a response to tracheal suction, endotracheal intubation, laryngoscopy or other tracheal stimuli are found mainly during the acute phase, in patients with high complete cervical SCI, and this problem usually resolves within the first 2–6 weeks after SCI. 

Cardiac arrhythmias are correlated with the level and severity of SCI.[18, 41–43] 

Autonomic cardiovascular control system influences heart rate through direct effects on the sinus node and by modulating circulating beta–adrenergic agonist levels. Heart rate variability establishes in a quantitative manner different shifts in autonomic control. The use of heart rate variability in SCI was reported.[41, 42–44] The validity of this noninvasive technique was not investigated in patients with efferent autonomic activity. Increasing in diminishing of both vagal and sympathetic activity is directly proportionate with the height and severity of the SCI.[41] Vagal activity influences both low frequency and high frequency components of heart rate variability during supine and upright postures. Sympathetic activity influences only the low frequency component of heart rate variability during upright position

In high and complete SCI there is a great reduction in both vagal and sympathetic activities, which indicates that the branches of the autonomic nervous system maintain a balance in the presence of SCI.[41]

Spontaneous beat–to–beat variability in R–R intervals in complete tetraplegic patients and in normal individuals was accomplished by autoregressive power spectral analysis. In healthy subjects two major spectral components were found, low and high frequency, while in tetraplegic patients only the high frequency component was noted. The authors conclude that the disappearance of the low frequency component in tetraplegic patients is due to the disruption of pathways from supraspinal cardiovascular centers to the peripheral sympathetic outflow, and that the cervical spinal sympathetic pathways may be instrumental in the genesis of the low frequency component in humans.[42] 

Variability of spontaneous beat–to–beat systolic blood pressure (Mayer waves) was investigated by means of autoregressive power spectral analysis. In tetraplegic patients the disappearance of the low frequency component in the systolic blood pressure variability (Mayer waves) is presumably caused by the interruption of the spinal pathways linking supraspinal cardiovascular centers with the peripheral sympathetic outflow and the cervical spinal sympathetic pathways may be instrumental in the genesis of the Mayer waves in humans.[45] 

Spontaneous beat–to–beat heart rate was investigated by autoregressive power spectral analysis. The majority of the tetraplegic patients had only the high frequency component with center frequency respiratory frequency, marker of vagal modulation of heart rate. In tetraplegic patients presenting both high and low frequency component, the center frequency of the low frequency component was lower than that in normal individuals, and the power of the high frequency component was smaller than normal.  The low frequency/high frequency power ratio, an index of sympatheticovagal balance, was larger than that in normal individuals The center frequency of the low frequency component had a center frequency of 0.03–0.15 Hz, and it is a marker of sympathetic and vagal modulation of heart rate. In tetraplegic patients with intact spinal cord at T1–T4, the level from where the cardiac sympathetic nerves originate, the total power, low frequency and high frequency components are smaller in normal subjects.[43] 

Vagal and sympathetic activity on heart rate variability can be investigated using pharmacological blockade. Atropine reduces amplitudes of both low (0.04–0.15 Hz) and high frequency (0.15–0.40 Hz) power spectrums during supine and upright postures. [18]  Propranolol reduces the low frequency amplitude with upright postures and has little effect on heart rate at rest.[18] 

Cardiovascular control studied in complete cervical SCI, in patients with recent SCI and previous spinal shock, and in patients with chronic SCI with reflex spinal cord activity was investigated by measuring blood pressure, heart rate, plasmatic levels of norepinephrine and epinephrine were measured at rest, during and after application of noxious stimuli below the level of lesion. Average resting blood pressure in tetraplegic patients with the recent SCI was 130/57mmHg (mean 81 mmHg), in the chronic SCI 107/55mmHg (mean 73 mmHg), compared with normal individuals   122/82 mmHg (mean 95 mmHg). 

Average heart rate at rest was 64 beats/minute in acute SCI, 73 beats/minute in chronic SCI and 77 beats/minute in healthy individuals.

In the acute SCI bladder stimulation causes minimal changes in heart rate and plasma norepinephrine and epinephrine levels. In the chronic SCI bladder stimulation, induce bradycardia and elevation in plasma norepinephrine but not in epinephrine levels. Cold stimuli in recent SCI did not change heart rate. Resting plasma norepinephrine and epinephrine levels in both the recent and chronic SCI were lower than in normal subjects.[46] 

Clinicians commonly use the head–up tilt to illuminate alterations in sympathetic cardiac modulation, which reflect the degree of autonomic dysfunction. Autonomic and cardiac responses to head–up tilt are blunted in both persons with tetraplegia and paraplegia.[47] In fact, persons with paraplegia had comparable increases in heart rate during head–up tilt as nondisabled persons, which were facilitated by the predominantly vagal withdrawal rather than the increased sympathetic activation that was demonstrated in nondisabled subjects. Heart rate variability techniques could be used to noninvasively assess cardiac autonomiccontrol in persons with SCI, determine the degree of sympathetic disruption, and illuminate the potential risk of developing cardiac dysrhythmias. During the acute rehabilitation period, heart rate variability can be a tool for monitoring improvements in autonomic outflow, and during the chronic phase of injury, it may document gains in function following physical and phar–macological interventions. SCI with resultant tetraplegia or high paraplegia is associated with significant dysfunction of the sympathetic nervous system. It occurs as a consequence of the loss of supraspinal control of the sympathetic nervous system. Below the level of injury occur reduced overall sympathetic activities, morphologic changes in sympathetic preganglionic neurons, and peripheral alpha–adrenoceptor hyperresponsiveness. Peripheral alpha–adrenoceptor hyperresponsiveness accounts for excessive pressor response in autonomic dysreflexia,decreased blood flow in the peripheral microcirculation, and increased susceptibility to pressure sores.

Alpha–adrenoceptor hyperresponsiveness may be a consequence of receptor hypersensitivity or a failure of presynaptic reuptake of norepinephrine at the receptor level.[13] Cardiovascular dysfunctions improve in time. The reasonsare not totally understood, but synaptic reorganization or hyperresponsiveness of alpha receptors may play a role in it. So far there no convincing data to demonstrated whether deafferented spinal cord can generate significant basal sympathetic activity. [14] 

#### Cardiovascular deconditioning

Cardiovascular deconditioning occurs as a consequence of prolonged bed rest and it is a loss of orthostatic tolerance, characterized by postural tachycardia and postural hypotension, thought to be related to diminished blood volume, decreased muscle or tissue pressure in the extremities or to functional alterations in the sympathetic nervous system.[48] Cardiovascular deconditioning is associated with altered nitric oxide metabolism.[2, 49,50] There is an higher production level of nitric oxide in the heart, brain, kidney, and systemic arteries, and a decreased nitric oxide production in the cerebral arteries. Altered nitric oxide metabolism it induces peripheral vasodilatation, with hypotension, and cerebral vasoconstriction with cerebral hypoperfusion. Increased release of nitric oxide and upregulation of nitric oxide synthase is associated with orthostatic intolerance after prolonged bed rest.[50].

Cardiovascular deconditioning is encountered in the acute phase, and its incidence is diminished with progression to the chronic phase, due to mobilization of the patient. 

### Cardiac dysfunctions during the chronic phase of SCI

#### Autonomic dysreflexia

After spinal shock resolution, the deafferented spinal cord, in SCI above T6, generates automonic dysreflexia, a life–threatening hypertensive bouts with compensatory bradycardia, after noxious stimuli or bladder or bowel distension. Autonomic dysreflexia is the result of the lack of supraspinal control of the sympathetic neurons and altered glutamatergic neurotransmission within the spinal cord.[14] 

Autonomic dysreflexia, observed for the first time by Anthony Bowlby in 1890, and described by Guttmann and Whitteridge in 1947 [51], is the consequence of interruption of control of the sympathetic spinal cord centers by central nervous centers, and it is a syndrome of massive imbalanced reflex sympathetic discharge to stimuli acting below the level of injury. Noxious stimuli produce disturbances in cardiac rhythm and profound alterations in sympathetic vasomotor, pilomotor and sudomotor activity. Distension, stimulation or manipulation of bladder or bowel are frequently determinant factors. [24,27,51– 54] 

Autonomic dysreflexia is a condition which may occur in individuals with SCI above the splanchnic sympathetic outflow, usually after cervical SCI. The prevalence varies between 48 and 90% of SCI above T6 [51, 55,56], but sometimes autonomic dysreflexia was encountered in SCI as low as T10 [51]. Morbidity is associated with the hypertension, which can cause retinal, subarachnoid or intracerebral hemorrhage, myocardial infarction, pulmonary edema or seizures. Mortality is rare. [51] Autonomic dysreflexia is characteristic for the chronic phase, but it may occur any time following SCI. It is rare within the first month following SCI. The incidence of precocious autonomic dysreflexia, occurring within the first month after SCI is 5.2%. [2,57, 68] 

After spinal shock resolution, the deafferented spinal cord, in injuries above T6, may generate aberrant impulses to noxious stimuli, leading to autonomic dysreflexia, a life–threatening complication of SCI. Autonomic dysreflexia is a syndrome of reflex sympathetic discharge from the preganglionic neurons in the thoracolumbar spinal cord occurring in patients with SCI located above the splanchnic sympathetic outflow T5–T6, often triggered by distension of pelvic viscera. Autonomic dysreflexia is a consequence of supraspinal control loss of sympathetic spinal cord neurons and altered glutamatergic neurotransmission within the spinal cord. [14] Viscerosensitive impulses, below the level of injury, are transmitted through intact peripheral sensory nerves, through ascendant pathways to neurons located within the intermediolateral thoracolumbar nuclei, releasing a sympathetic reflex. Sympathetic hyperstimulation discharge high quantities of norepinephrine, dopamin–beta–hydroxilasis and dopamine, which leads to massive vasoconstriction within arterial system, arterial hypertension, and cerebral vasodilatation. The brain perceives the hypertensive crisis throughout cervical baroreceptors and 9 and 10 nerves. It generates inhibitory impulses that cannot be transmitted below the level of injury. Vasomotor centers from the medulla oblongata tries to lower arterial blood pressure, by parasympathetic stimulation of the heart, through 10 nerve, generating severe bradycardia.

The development of autonomic dysreflexia is correlated with aberrant sprouting of peptidergic afferent fibers into the spinal cord below the injury. Sprouting of nerve growth factor–responsive afferent fibers has been shown to have a major influence on dysreflexia, perhaps by amplifying the activation of disinhibited sympathetic neurons.[59] 

Peripheral alpha–adrenoreceptor hyperresponsiveness following SCI, and this may play a significant role in the development of autonomic dysreflexia.[2] 

There is a constellation of signs and symptoms in SCI above T5–T6 in response to noxious or nonnoxious stimuli below injury level. Usually, cardiac response encountered during autonomic dysreflexia is reflex bradycardia. But autonomic dysreflexia may present also with recurrent ventricular fibrillation and cardiac arrest, as a result of profound effects of massive paroxysmal sympathetic activity associated with this condition.[52] Other clinical features includes severe arterial hypertension, headache and visual impairment due to cerebral vasodilatation, cutaneous pallor below the injury site, piloerection, secondary to sympathetic activity, profuse sweating and cutaneous vasodilatation above the level of lesion, secondary to parasympathetic activity. Arterial blood pressure can reach up to 300 mmHg, leading to retinal, intracerebral, or subarachnoid hemorrhage, pulmonary edema, myocardial infarction, seizures, confusion and death.[27, 60] According to increasing blood pressure, there are three severity grades: mild/partial, when blood pressure increases with less than 40 mmHg, moderate, when blood pressure increases with more than 40 mmHg, but systolic blood pressure is under 180 mmHg, and severe, when systolic blood pressure increases over 180 mmHg.

#### Coronary heart disease

Coronary heart disease accounts for approximately 20% of deaths in SCI population. This risk of coronary heart disease may be increasingly important as the life expectancy of people with SCI lengthens.

Coronary heart disease increases after SCI due to physical inactivity, obesity, hyperlipidemia, insulin resistance, and diabetes. Abnormal lipid profile, with increasing in total cholesterol and low–density lipoprotein (LDL–cholesterol) levels and diminishing of the high–density lipoprotein (HDL–cholesterol) level occur in chronic SCI. The reason for diminishing of the HDL–cholesterol is still on debate. It is assumed that it due to inappropriate diet, adrenergic dysfunction and lack of physical activity.[1] The ratio of total cholesterol/HDL–cholesterol > 5 is considered high risk for coronary heart disease.[1] 

## Treatment

### Treatment of neurogenic shock

Treatment of neurogenic shock implies correction of bradycardia and hypotension. As a first step two venous lines should be established for administration of resuscitation fluids and medications. 

The treatment of neurogenic shock is individualized for each patient, and depends on the severity of heart rate dysfunctions and blood pressure. 

Hypotension, associated with normal heart rate requires volume loading, with crystalloids and colloids within the first 24–48 hours following SCI. If there is no concomitant hypovolemic shock, Hartmann's solution is administrated 50–100 ml per hour, in order maintain a systolic blood pressure > 80 mmHg. If besides neurogenic shock there is an associated hypovolemic shock, normal saline or Hartmann’s solution is administrated with a rate big enough to increase intravascular volume and blood pressure.

Patients having bradycardia with heart rate < 60/minute and hypotension with systolic blood pressure < 90 mmHg, require 0.5 mg atropine for low heart rate and sympathomimetics for hypotension, such as ephedrine, administrated intravenous 5 mg or subcutaneously 25–50 mg, every 4–6 hours. 

Heart rate < 50/minute, nodal or ventricular ‘escapex2019; dysarrhythmias require higher doses of atropine administered as often as necessary up to 2 mg per hour. 

In severe neurogenic shock, monitoring of the central venous pressure is mandatory in order to ensure fluid load up to a central venous pressure 7–10 mmH2O. If hemodynamic instability persists, a Swan Ganz catheter is assembled. It provides accurate information on cardiac output, heart preload and systemic vascular resistance. In cases with profound hemodynamic instability with low central venous pressure, large doses of vasopressors, like norepinephrine, dobutamine and dopamine are required. 

Tisular perfusion must be maintained. Hypotension, consequence of neurogenic and/or hypovolemic shock causes spinal cord ischemia, cord damage and extending of neurological deficit. In tetraplegic patients blood pressure must be maintained ranging from 80/40 mmHg to 100/60 mmHg. Attention should be paid to fluid overload that can cause cord edema, which will further reduce tisular perfusion. 

### Treatment of bradyarrhythmias

Treatment of bradyarrhythmias consist of prophylactic measures, curative treatment, and cardiac rehabilitation. 

In SCI patients, monitoring heart rate is mandatory, and, if indicated, providing support with medications to increase heart rate and blood pressure.

The first line therapy for bradycardia is dopamine administration. Atropine and transcutaneous pacer are used as the second line therapy in cases that do not respond to dopamine administration. Transvenous pacer is necessary in prolonged or excessive bradycardia. The last treatment resource is permanent pacing, but it is rarely necessary. Aminophylline and methylxanthines given in patients with SCI and low heart rate were associated with resolution of the bradycardia. They were used to prevent episodic bradyarrhythmias after SCI.[ 61] 

Prevention of bradycardia and cardiac arrest includes adequate oxygenation and atropine administration before tracheal suction, laringoscopy or other maneuver that stimulate the trachea in all artificially ventilated tetraplegic patients. [32,33,40] Full lung expansion before suctioning may decrease vagal tone, therefore, provide a full breath with a ventilator or bag–valve–mask resuscitator (Ambu–bag).

Rehabilitation program includes physical therapy. Early management begins with assessment of potential exacerbating factors, including prolonged recumbency, rapid changes in positioning, underlying infection, dehydration, and drug adverse effects. Before moving the patient out of supine position, abdominal binderm, thigh–high antiembolism stockings, and elastic bandages to the lower extremities are applied. These measures decrease venous pooling in the lower extremities and splanchnic vasculature. The patient is moved slowly, from a supine position to a relatively upright position. Using a tilt table which slowly increases in degrees of tilt, can help the patient acclimate to an upright position. During the period of acclimatization, active arm exercises are useful to maintain blood pressure while the patient is on the tilt table.[1]

Bilaterally functional electric stimulation to lower–limb muscles, quadriceps, hamstrings, tibialis anterior and gastrocnemius, at an intensity that provides a strong, visible, and palpable contraction during postural tilting improves orthostatic tolerance in patients with cervical complete motor SCI. Functional electric muscle stimulation during tilting maneuver, significantly increase heart rate by 1.0+/–0.5 beats/minute, and attenuated the drop in systolic and diastolic blood pressure for every 15 degrees increment in the angle of the tilt, and by this increasing the overall mean standing.[62]

Other exercises that increase cardiac fitness are electrically assisted exercises for leg cycling, arm ergometry and ambulation. Cardiovascular deconditioning is reversible by training by increasing vascular resistance in paralyzed legs. [63] 

Cardiac rehabilitation requires special consideration of patient's inability to tolerate traditional antianginal medications, because of low blood pressures, and adaptations, progressive wheelchair propulsion, to address their limitations in mobility. 

### Treatment of associated orthostatic hypotension

Treatment of associated orthostatic hypotension can be made through nonpharmacological and pharmacological methods. Mean arterial pressure of at least 85 mmHg is necessary to maintain spinal–cord perfusion and help prevent secondary ischemia.

Nonpharmacologic measures includes avoidance of precipitating factors of orthostathic hypotension, such as diuretics, alcohol, caffeine and vasodilatory stresses [64–66], maintenance of plasma volume by increased salt and fluid intake [64,65], serving regular small meals that minimize postprandial hypotension [2, 64,66], wearing compression bandages and/or support stockings which restrict venous pooling in the splanchnic region and limbs [2, 66] and maintenance of elevated position of the head with 10–20^Ŷ^, during the night, this maneuver being known to increase plasma volume and orthostatic tolerance[67]. Patients should be advice about symptoms of orthostatic hypotension and they should be encouraged to assume a recumbent or semirecumbent position if they occur. [64] 

Pharmacological therapy includes plasma volume expansion with fludrocortisones [68] or increasing peripheral vasoconstriction with the alpha–adrenergic agonist midodrine. [69,70] In patients with more refractory symptoms, desmopressin acetate or erythropoietin can be used. [2] Nitric oxide synthase inhibitors are currently under investigation, and results seem promising. They normalize supine blood pressure in SCI with tetraplegia. [50] Peripheral alpha–adrenoceptor hyperresponsiveness potentially increases susceptibility to pressure sores. 

### Treatment of deep vein thrombosis

Prophylaxis of deep vein thrombosis is mandatory through nonpharmacologic prophylactic strategies including early mobilization of the patient, thigh high compression stockings, pneumatic calf compression boots and physical therapy and pharmacologic prophylactic treatment consisting of anticoagulant therapy, such as heparin, low molecular weight heparin or oral anticoagulants. 

Heparin administered subcutaneously is useful for reducing blood viscosity and improving flow. Low molecular weight heparin has been demonstrated to be superior to standard heparin preparations and the former also significantly reduces the incidence of bleeding. Because of the high risk of developing deep vein thrombosis, in patients with SCI regular measurement of bilateral calf and thigh circumferences is mandatory. Doppler ultrasound or venograms are frequently performed to establish baselines.

Curative treatment for deep vein thrombosis is represented by intravenous heparin for 7 to 10 days, and once adequate anticoagulation is provided, oral anticoagulant therapy for 3 months.

In patients in whom thromboembolism occurs under anticoagulation therapy or for patients with high risk to anticoagulant therapy, an inferior vena cava filter can be placed.

### Treatment of autonomic dysreflexia

Avoidance of trigger factors like noxious stimuli, bowel or bladder stimulation or distension prevents the occurrence of autonomic dysreflexia. Sympatholitic drugs administration (alpha–adrenergic blocking agents, ganglionic blockers, catecholamine depleters), vasodilators drugs and local anesthetics can prevent onset of this phenomenon. [55, 71,72] Oral terazosin is administrated prophylactic. Treatment of autonomic dysreflexia consists of elevated position of head and trunk and sublingual administration of nifedipine or nitroglycerine for immediate effect. Other drugs used are mecamylamine, diazoxide, and phenoxybenzamine. [51] The main therapeutic issue is removal trigger factors, bladder and bowel decompression, because persistence of visceral stimuli maintain sympathetic response. [27] Analgesia is obtained by administration of paracetamol or co–proxamol, aspirin or non–steroidal anti–inflammatory drugs being contraindicated. [24] Unresponsive patients or with poor response to therapy are indicated for regional or general anesthesia, that successfully blocks sympathetic response. [56]  

### Treatment of coronary heart disease and dyslipidemia

Coronary heart disease prevention is based on avoidance of risk factors, including high blood pressure, smoking, obesity, physical inactivity, and cholesterol and/or lipid control.

Lipid–lowering drugs are used used for dyslipidemia. Goals for optimal cholesterol management currently include an LDL–cholesterol level of < 100 mg/dl, and total cholesterol of < 200 mg/dl.1

Resistive exercise therapy can improve arterial health after chronic SCI, which may reduce the risk of coronary heart disease. Aerobic exercise improves lipid profiles in patients with paraplegia but they less effect in patients with tetraplegia. 

### Treatment of associated pathology

Associated pathology, such as type 2 diabetes mellitus, progressive renal and cardiovascular disease often requires additional treatment. Angiotensin–converting enzyme inhibitors are often necessary for progressive renal and cardiovascular disease. Tetraplegic patients are tolerant of an acute bout of orthostatic stress after partial angiotensin–converting enzyme inhibitors administration. Although mean arterial blood pressure, is reduced immediately after angiotensin–converting enzyme inhibitors administration, it is after that maintained with increasing angles of tilt and no symptoms occurred. [47]

### Pharmacological interactions

Succinylcholine must be avoided in patients with SCI because it may induce cardiac arrest and hypokalemia as a result of hypersensitivity of muscle–cell membranes. Other medications that should be avoided include antihypertensives, diuretics, tricyclic antidepressants, anticholinergics, narcotic analgesics, and sildenafil.

## Conclusions

Cardiac dysfunctions are common consequences following SCI. cardiovascular disturbances are the leading causes of morbidity and mortality in both acute and chronic phases of SCI. Disruption of descendent pathways from superior centers to spinal sympathetic neurons, originating into the intermediolateral nuclei of T1–L2 spinal cord segments results in an reduced overall sympathetic activity and unopposed parasympathetic tone through intact vagus nerve. Impairment of autonomic nervous control system in patients with high SCI, cervical and high–thoracic, causes cardiac dysrrhythmias. The most common cardiac dysrrhythmias are bradyarrhythmias, bradycardia and rarely cardiac arrest. Tachyarrhythmias, such as paroxistic supraventricular tachycardia, sinus tachycardia, atria flutter and atria fibrillation, are less frequently encountered. Spinal shock, with neurogenic shock is encountered during the acute phase, while autonomic dysreflexia is found in the chronic phase, after spinal shock resolution. Cardiac dysfunction may be avoided, treated or deceased by proper prophylactic measures, curative treatment and rehabilitation.
